# Isolation of Acetylated and Unmodified Protein N-Terminal Peptides by Strong Cation Exchange Chromatographic Separation of TrypN-Digested Peptides

**DOI:** 10.1074/mcp.TIR120.002148

**Published:** 2020-11-24

**Authors:** Chih-Hsiang Chang, Hsin-Yi Chang, Juri Rappsilber, Yasushi Ishihama

**Affiliations:** 1 Graduate School of Pharmaceutical Sciences, Kyoto University, Kyoto, Japan; 2 Graduate Institute of Metabolism and Obesity Sciences, Taipei Medical University, Taipei, Taiwan; 3 Bioanalytics, Institute of Biotechnology, Technische Universität Berlin, Berlin, Germany; 4 Wellcome Centre for Cell Biology, School of Biological Sciences, University of Edinburgh, Edinburgh, United Kingdom; 5 Laboratory of Clinical and Analytical Chemistry, National Institute of Biomedical Innovation, Health and Nutrition, Ibaraki, Osaka, Japan

**Keywords:** protein N terminus, N terminomics, TrypN, SCX, N-terminal peptide enrichment, acetylated N-terminal peptide, LC/MS/MS, shotgun proteomics, HEK293T, ACN, acetonitrile, CAA, 2-chloroacetamide, ChaFRADIC, charge-based fractional diagonal chromatography, COFRADIC, combined fractional diagonal chromatography, HYTANE, hydrophobic tagging-assisted N-termini enrichment, MS, mass spectrometry, PTS, phase-transfer surfactants, SCX, strong cation exchange chromatography, SDC, sodium deoxycholate, SLS, sodium N-lauroylsarcosinate, StageTip, stop and go extraction tip, TAILS, terminal amine isotopic labeling of substrates, TCEP, tris(2-carboxyethyl)phosphine, TFA, trifluoroacetic acid, Tris-HCl, tris(hydroxymethyl)aminomethane hydrochloride

## Abstract

We developed a simple and rapid method to enrich protein N-terminal peptides, in which the protease TrypN is first employed to generate protein N-terminal peptides without Lys or Arg and internal peptides with two positive charges at their N termini, and then, the N-terminal peptides with or without N-acetylation are separated from the internal peptides by strong cation exchange chromatography according to a retention model based on the charge/orientation of peptides. This approach was applied to 20 μg of human HEK293T cell lysate proteins to profile the N-terminal proteome. On average, 1550 acetylated and 200 unmodified protein N-terminal peptides were successfully identified in a single LC/MS/MS run with less than 3% contamination with internal peptides, even when we accepted only canonical protein N termini registered in the Swiss-Prot database. Because this method involves only two steps, protein digestion and chromatographic separation, without the need for tedious chemical reactions, it should be useful for comprehensive profiling of protein N termini, including proteoforms with neo-N termini.

## Highlights

• Isolation of protein N-terminal peptides without chemical reaction
• Both N-acetylated and unmodified protein N termini can be isolated
• Two-step isolation consisting of TrypN digestion and strong cation exchange separation
• Less than 3% contamination with internal peptides

## Graphical Abstract

**Figure:**
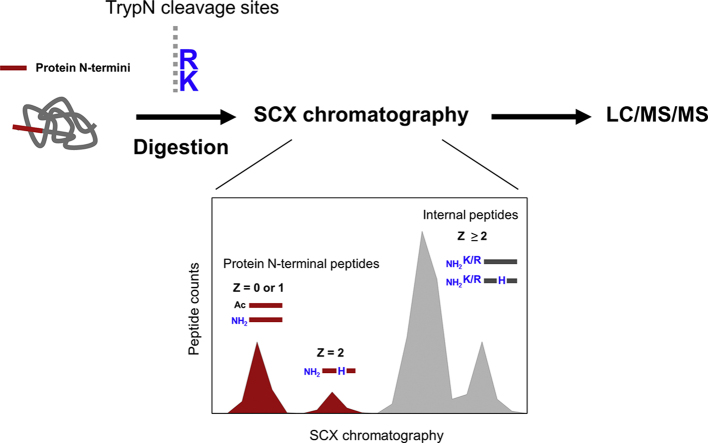

## In Brief

N-acetylated and unmodified protein N-terminal peptides were successfully isolated by strong cation exchange chromatographic separation of TrypN-digested peptides without chemical reaction. From 20 μg of human HEK293T cell lysate proteins, 1550 acetylated and 200 unmodified protein N-terminal peptides were identified in a single LC/MS/MS run with less than 3% contamination with internal peptides, even when we accepted only canonical protein N termini registered in the Swiss-Prot database.

Characterizing protein N termini is essential to understanding how the entire proteome is generated through biological processes such as translational initiation (1, 2, 3), posttranslational modifications (4, 5), and proteolytic cleavages (6, 7). To perform N terminomics using MS, peptides derived from protein N termini must be selectively enriched, and many methods have been developed for this purpose (8, 9). Some of them use “positive selection” approaches in which chemically labeled protein N-terminal peptides are enriched by affinity purification (6, 10). However, these approaches are not applicable to proteins with *in vivo* N-terminal modifications. In contrast, “negative selection” approaches to isolate protein N-terminal peptides by depleting internal peptides have been used to comprehensively identify protein N-terminal peptides, including N-terminal modifications such as methylation, acetylation, and lipidation (11, 12). Gevaert *et al.* pioneered combined fractional diagonal chromatography (13), and this was followed by other negative selection approaches such as terminal amine isotopic labeling of substrates (14), the variant of combined fractional diagonal chromatography called charge-based fractional diagonal chromatography (15), and hydrophobic tagging-assisted N-terminal enrichment (16). All of them require blocking of the primary amines at the protein level and depletion of digested internal peptides by means of chemical tagging–based separation. Thus, relatively large amounts of samples (∼5–10 mg) are generally required to increase the identification number of protein N-terminal peptides. This limits the usefulness of these approaches in the case of hard-to-obtain biological samples (17, 18). Furthermore, limitations in the efficiency and specificity of the chemical derivatizations compromise the confidence of peptide identification. Therefore, a simple and sensitive approach to enrich protein N-terminal peptides is still needed for MS-based proteomics.

Strong cation exchange (SCX) chromatography, employing Coulombic interactions to separate peptides based on their charge at acidic pH, has been widely applied for deep proteomic profiling (19, 20). Alpert *et al.* (21) reported that the peptide retention in SCX is affected by charge and orientation. In SCX separation of tryptic peptides, acetylated protein N-terminal peptides and protein C-terminal peptides are eluted first. Monophosphorylated peptides with a +1 charge are then eluted, followed by peptides with a +2 or more charge, such as unmodified protein N-terminal peptides, internal peptides, and peptides containing missed cleavages (22, 23). Thus, it is impossible to isolate protein N-terminal peptides from tryptic peptides by SCX chromatography. This is also the case when Lys-N was used with SCX chromatography, based on the charge/orientation retention model (21). To overcome this issue, we focused on TrypN, also known as LysargiNase, a metalloprotease that cleaves peptide chains mainly at the N-terminal side of Lys/Arg even in the case of Pro-Lys and Pro-Arg bonds, generating peptides with N-terminal Lys/Arg and yielding protein N-terminal peptides that do not contain Lys/Arg (24). Unlike other kinds of LysargiNase such as ulilysin (25, 26) and mirolysin (27), which preferentially cleave the N-terminal side of either Lys or Arg, TrypN cleaves the N-terminal side of Lys and Arg equally at pH 6∼8. Moreover, the peptide identification performance for N-terminal Lys/Arg peptides is comparable to that for tryptic peptides (28).

In this study, we developed a new method to enrich protein N-terminal peptides without the need for chemical derivatization or complex procedures, taking advantage of the combination of proteinase TrypN-mediated protein cleavage and SCX separation of N-terminal peptides based on the extended charge/orientation retention model. We show that this rapid and simple approach to enrich protein N-terminal peptides enables comprehensive, high-throughput analysis of the human and bacteria N-terminal proteomes.

## Experimental Procedures

### Materials

Ammonium bicarbonate, tris(hydroxymethyl)aminomethane hydrochloride, SDC, sodium N-lauroylsarcosinate, ammonium bicarbonate, tris(2-carboxyethyl)phosphine, 2-chloroacetamide, calcium chloride, ethyl acetate, acetonitrile (ACN), acetic acid, TFA, and other chemicals were purchased from Fujifilm Wako. RapiGest was purchased from Waters Corporation. Modified trypsin was from Promega. TrypN was from Protifi. Styrene divinylbenzene (SDB-XC) Empore disk was purchased from GL Sciences. Water was purified by a Millipore Milli-Q system.

### Cell Culture and Protein Extraction

HEK293T (human embryonic kidney) cells were cultured to 80% confluence in 10-cm diameter dishes. *E. coli* K-12 BW25113 cells were grown to mid-log phase in LB broth with vigorous shaking at 37 °C. These cells were collected by centrifugation and resuspended in the PTS lysis buffer containing protease inhibitors (Sigma), 12 mM SDC, 12 mM sodium N-lauroylsarcosinate, 10 mM tris(2-carboxyethyl)phosphine, 40 mM 2-chloroacetamide in 100 mM Tris buffer (pH 8.5) (29, 30). The lysate was vortexed and sonicated on ice for 20 min. The concentration of protein crude extract was determined by means of bicinchoninic acid protein assay (ThermoFisher Scientific).

### Protein Digestion

For optimization of TrypN digestion conditions, protein pellets were prepared by methanol/chloroform precipitation as described previously (31) and were dissolved with 0.1% RaipGest in the buffer consisting of 25 mM trimethylammonium acetate, 2 mM CaCl, and 0.1 mM MnCl_2_ at pH 7.4, followed by TrypN digestion overnight at 55 °C according to the manufacturer's protocol. The PTS buffer (29) or the urea buffer consisting of 1 M urea, 25 mM trimethylammonium acetate, 2 mM CaCl, and 0.1 mM MnCl_2_ at pH 7.4, instead of the RapiGest buffer was also used for the TrypN digestion.

For TrypN digestion after optimization, the cell lysate in the PTS buffer was diluted ten-fold with 10 mM CaCl_2_ and digested with TrypN (1: 50 w/w) overnight at 37 °C. Note that TrypN can be replaced with LysargiNase (Merck Millipore). In the case of tryptic digestion, the protein solution was digested with Lys-C (1:50 w/w) for 3 h at 37 °C, followed by five-fold dilution with 50 mM ammonium bicarbonate and trypsin digestion (1:50 w/w) overnight at 37 °C. After enzymatic digestion, an equal volume of ethyl acetate was added to each sample solution, and the mixture was acidified with 0.5% TFA (final concentration) according to the PTS protocol reported previously (29). The resulting mixture was shaken for 1 min and centrifuged at 15,700*g* for 2 min to separate the ethyl acetate layer. The aqueous layer was collected and desalted by using StageTips with SDB-XC disk membranes (SDB-StageTip) (32). The proteolytic peptides were quantified by LC-UV at 214 nm using bovine serum albumin digest as a standard and kept in 80% ACN and 0.5% TFA at −20 °C until use.

### Peptide Fractionation by SCX HPLC

SCX chromatography was performed using a Prominence HPLC system (Shimadzu) with a BioIEX SCX column (250 mm × 4.6 mm inner diameter, 5 μm nonporous beads made of poly[styrene-divinylbenzene] modified with sulfonate groups) (Agilent).

For examination of the SCX separation characteristics, 75 μg each of trypsin- and TrypN-digested HEK293T peptides were mixed and directly loaded onto the SCX column at 0.8 ml/min. A mixture of 5 mM potassium phosphate (pH 3.0) and ACN (7:3) was used as SCX buffer A, and a mixture of 500 mM potassium phosphate (pH 3.0) and ACN (7:3) was used as SCX buffer B. Gradient elution was performed as follows: 0% B for 5 min, 0 to 10% in 20 min, 10 to 50% in 10 min, 50 to 100% in 5 min, and 100% B for 4 min. Fractions were manually collected at 1-min intervals for 45 min. After evaporation of the solvent in a SpeedVac SPD121P (ThermoFisher Scientific), fractionated peptides were resuspended in 50 μl of 0.1% TFA and desalted by using SDB-StageTips. One-fourth of each fraction was analyzed by nanoLC/MS/MS using a TripleTOF 5600 (SCIEX) as described below.

For gradient SCX fractionation of TrypN-digested HEK293T peptides, 80 μg of digested peptides were analyzed using the SCX HPLC system described above. A mixture of 7.5 mM potassium phosphate (pH 2.6) and ACN (7:3) was used as SCX buffer A, and 350 mM KCl was added to buffer A for SCX buffer B. Gradient elution was performed as follows: 0.5% B for 15 min, 0.5 to 1% B in 10 min, 1 to 4% B in 10 min, 4 to 10% B in 3 min, 10 to 100% B in 3 min, and 100% B for 5 min. Fractions were manually collected at 1-min intervals for 50 min. The fractionated peptides desalted by using SDB-StageTips as described above. One-fourth of each fraction for Fr.1 to 43 and one-tenth of each fraction for Fr.44 to 50 were analyzed by nanoLC/MS/MS using an Orbitrap Fusion Lumos mass spectrometer (ThermoFisher Scientific) as described below.

### Enrichment of Protein N-Terminal Peptides by SCX HPLC With Isocratic Elution

Enrichment of protein N-terminal peptides from 30 μg of TrypN-digested *E. coli* peptides was performed using the SCX HPLC system under the following isocratic conditions: SCX buffer A was a mixture of 7.5 mM potassium phosphate solution (pH 2.2) containing 10, 12.5, or 15 mM KCl and ACN (7:3), and buffer B was a mixture of 7.5 mM potassium phosphate solution (pH 2.2) containing 500 mM KCl and ACN (7:3). Isocratic elution was performed with 100% A for 30 min, and then the system was washed with 100% B. The collected fractions were lyophilized, resuspended in 50 μl of 0.1% TFA, and desalted using SDB-StageTips. Two-thirds of the enriched peptides were analyzed by nanoLC/MS/MS using the Orbitrap Fusion Lumos.

To isolate protein N-terminal peptides from TrypN-digested HEK293T peptides, the digested peptides (80 μg) were analyzed by the SCX HPLC system under isocratic conditions, eluting with a mixture of 7.5 mM potassium phosphate solution (pH 2.2) containing 10 mM KCl and ACN (7:3) for 30 min to collect the desired fraction and desalted using SDB-StageTips as described above. We analyzed one-fourth of the enriched peptides by nanoLC/MS/MS in triplicate using the Orbitrap Fusion Lumos.

### NanoLC/MS/MS Analysis

NanoLC/MS/MS analyses were performed on a TripleTOF 5600 mass spectrometer or an Orbitrap Fusion LUMOS mass spectrometer, connected to a Thermo Ultimate 3000 pump and an HTC-PAL autosampler (CTC Analytics). Peptides were separated on self-pulled needle columns (150 mm length × 100 μm ID, 6 μm opening) packed with Reprosil-Pur 120 C18-AQ 3 μm reversed-phase material (Dr. Maisch). The injection volume was 5 μl, and the flow rate was 500 nl/min. The mobile phases were (A) 0.5% acetic acid and (B) 0.5% acetic acid and 80% ACN. For TripleTOF 5600 analysis, gradient elution was performed as follows: 12 to 40% B in 20 min, 45 to 100% B in 1 min, and 100% B for 5 min. For Orbitrap analysis, gradient elution of fractionated samples was performed as follows: 12 to 40% B in 15 min, 40 to 100% B in 1 min, and 100% B for 5 min. For protein N-terminal peptide-enriched samples, gradient elution was performed as follows: 10 to 40% B in 100 min, 40 to 100% B in 10 min, and 100% B for 10 min. Spray voltages of 2300 V in the TripleTOF 5600 system and 2400 V in the Orbitrap system were applied. The mass scan range of the TripleTOF 5600 system was *m/z* 300 to 1500, and the top ten precursor ions were selected in each MS scan for subsequent MS/MS scans. The mass scan range for the Orbitrap system was *m/z* 300 to 1500, with an automatic gain control value of 1.00e + 06, a maximum injection time of 50 ms, and detection at a mass resolution of 60,000 at *m/z* 200 in the orbitrap analyzer. The top ten precursor ions with +2, +3, or +4 charge were selected in each MS scan for subsequent MS/MS scans with an automatic gain control value of 5.00e + 04 and a maximum injection time of 300 ms. Dynamic exclusion was set for 25 s with a 10 ppm gate. The normalized higher energy collisional dissociation was set to be 30, with detection at a mass resolution of 15,000 at *m/z* 200 in the Orbitrap analyzer. A lock mass (445.1200025) function was used to obtain constant mass accuracy during the gradient.

### Proteomics Data Processing

Two peak lists in “.mgf” and “.apl” formats were generated from the MS/MS spectra by MaxQuant 1.5.8.0 (33). The peptides and proteins were identified by Mascot v2.6.1 (Matrix Science) against the Swiss-Prot database (version 2017_4, 20,199 sequences) or the *E. coli* K-12 MG1665 protein sequence database (34) with a precursor mass tolerance of 20 ppm (TripleTOF 5600) or 10 ppm (Orbitrap), a fragment ion mass tolerance of 0.1 Da (TripleTOF 5600) or 20 ppm (Orbitrap), TrypN/trypsin specificity allowing for up to four missed cleavages for TrypN/trypsin mixed proteolytic peptides, and strict TrypN specificity allowing for up to two missed cleavages for TrypN-digested peptides. Carbamidomethylation of cysteine was set as a fixed modification, and methionine oxidation and protein N-terminal acetylation were allowed as variable modifications. False discovery rates at a peptide level of less than 1% were applied for peptide identification based on a target-decoy approach.

## Results and Discussion

### Retention Behavior of TrypN-Digested Peptides in SCX Chromatography

Proteolysis with TrypN yields peptides with at least a +2 charge with Lys or Arg and an α-amino group at the peptide N terminus at the acidic pH. By contreast, peptides derived from protein N termini have neither Lys nor Arg and are often acetylated at the protein N terminus so that most of them have a 0 or +1 charge, and only His-containing peptides with an unmodified protein N terminus have a +2 charge (supplemental Fig. S1). In this study, we focused on the fact that SCX chromatography under the acidic conditions might be able to separate peptides based on the number of positive charges as well as the localization of the charges according to the charge/orientation retention model (21), and we attempted to separate protein N-terminal peptides from internal peptides among TrypN-digested peptides.

We first examined the number of missed cleavages in TrypN digestion. When digestion was performed in 0.1% RapiGest according to the manufacturer's protocol, the missed cleavage rate (the content of peptides with two or more missed cleavage sites) was 14%, almost equal to the value in the condition without addition of RapiGest (16%). However, when 1% sodium deoxycholate (SDC) was added instead of RapiGest, the missed cleavage rate was dramatically reduced to 5.8%. Thus, TrypN digestion was performed according to the phase-transfer surfactants (PTS) protocol (29) in this study.

**Fig. 1:**
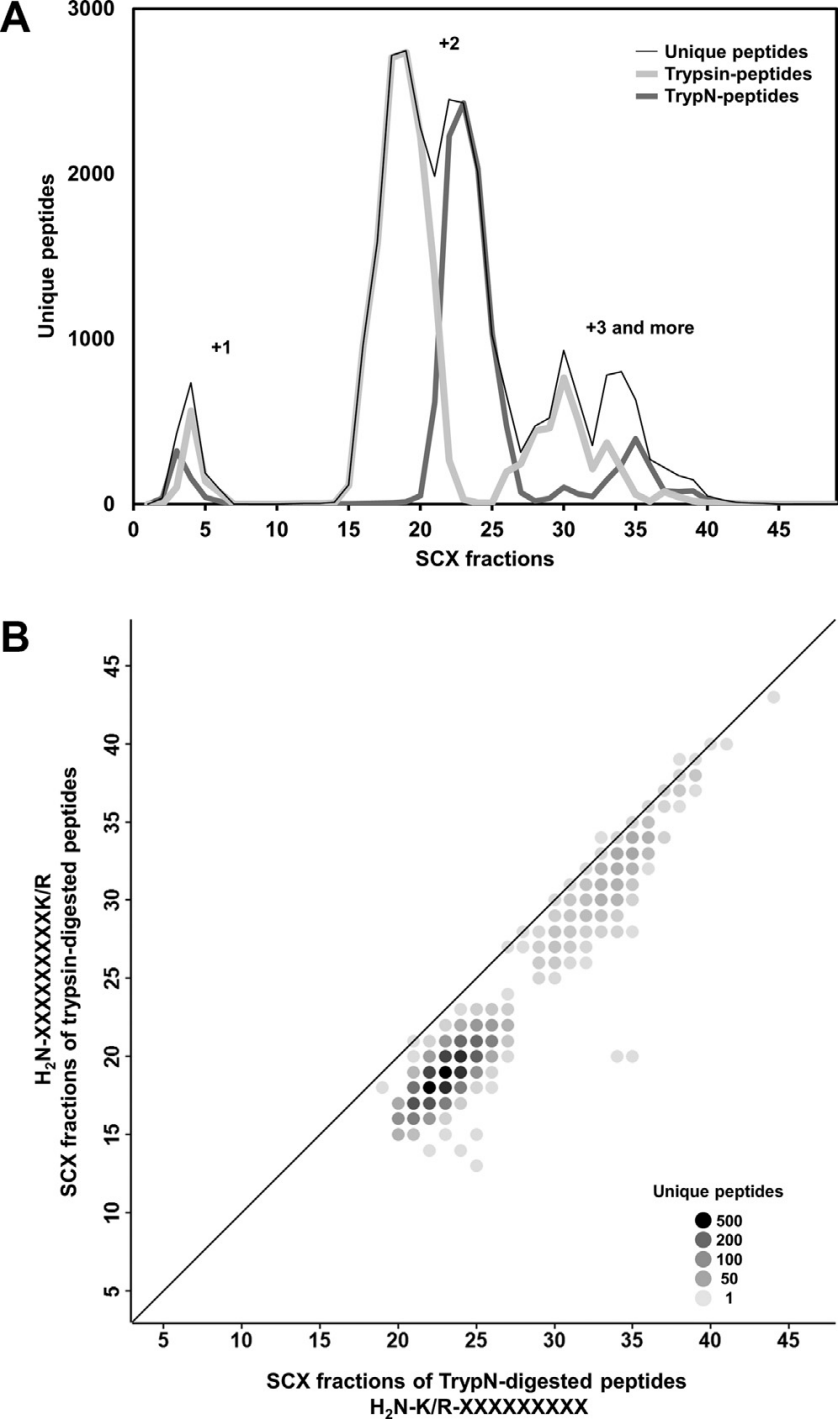
**SCX separation of TrypN-digested and trypsin-digested peptides**. *A*, SCX separation of different types of peptides in digested HEK293T cell lysate. The peptide sample was prepared by mixing TrypN- and trypsin-digested HEK293T peptides with 1:1. The *black*, *light gray*, and *dark gray curves* represent unique peptides, trypsin-digested peptides, and TrypN-digested peptides, respectively. The charge number at acidic pH was labeled. *B*, comparison of SCX elution profiles of TrypN- and trypsin-digested peptides. Peptides with the same sequence except for the termini were selected (K/R-XXXXXX and XXXXXX-K/R for TrypN- and trypsin-digested peptides, respectively). The shade of the circle color indicates the number of peptides. SCX, strong cation exchange.

Next, keeping in mind the need to separate protein N-terminal–derived peptides with both His residues and unmodified N termini from TrypN-digested internal peptides, we investigated whether the peptides could be separated based on the position of the positive charge, in addition to the number of positive charges, by SCX chromatography. Studies with proteases that cleave either Lys/Arg, such as Lys-C and Lys-N or trypsin and TrypN, have indicated that the position of the positive charge affects the outcome in shotgun proteomics (26, 35). For example, it has been reported that peptides with N-terminal Lys or Lys/Arg are more strongly retained than peptides with a C-terminal Lys or Lys/Arg in reversed-phase LC (28). To determine how the Lys/Arg position of peptides affects their retention behavior in SCX chromatography at acidic pH, we examined a mixture of TrypN- and trypsin-digested peptides using the SCX HPLC system, followed by nanoLC/MS/MS. The 19,853 unique tryptic peptides generally showed weaker retention than the 11,334 unique TrypN peptides with the same charge states (Fig. 1*A*, supplemental Table S1). To characterize the SCX elution profiles in more detail, we compared the retention time in SCX HPLC for approximately 4000 peptide pairs having sequences that differ only in the position of terminal Lys/Arg (Fig. 1*B*). As expected, TrypN-digested peptides exhibit stronger SCX retention than Trypsin analogs. This would be because the TrypN peptides carry two positively charged groups at the N terminus, because of the α-amino group of the N-terminal Lys/Arg and the side-chain ε-amino or guanidino group, whereas the positive charge of the C-terminal Lys/Arg of trypsin peptides was partially neutralized by the α-carboxy group (supplemental Fig. S1). Alpert *et al.* (21) and Gauci *et al.* (23) reported that Lys-N–digested phosphopeptides with two basic moieties in close proximity tend to be more strongly retained on an SCX column than tryptic phosphopeptides. Gussakovsky *et al.* (36) reported a retention model for predicting the retention times in SCX chromatography of tryptic peptides, in which the position-dependent coefficient of basic amino acids is higher near the N terminus. We also found that the TrypN-digested peptides eluted in a narrower SCX fraction range than the tryptic peptides (Fig. 1*A*). This may be because of the fact that the distance between N-terminal positive charge in the tryptic peptides differs depending on the length of peptide, whereas the TrypN-digested peptide has the N-terminal Lys/Arg that minimizes the distance between the two positive charges. To our knowledge, the present work is the first to validate the peptide charge/orientation retention model in SCX using thousands of identical sequence pairs.

### SCX HPLC Separation of TrypN-Digested HEK293T Peptides

**Fig. 2:**
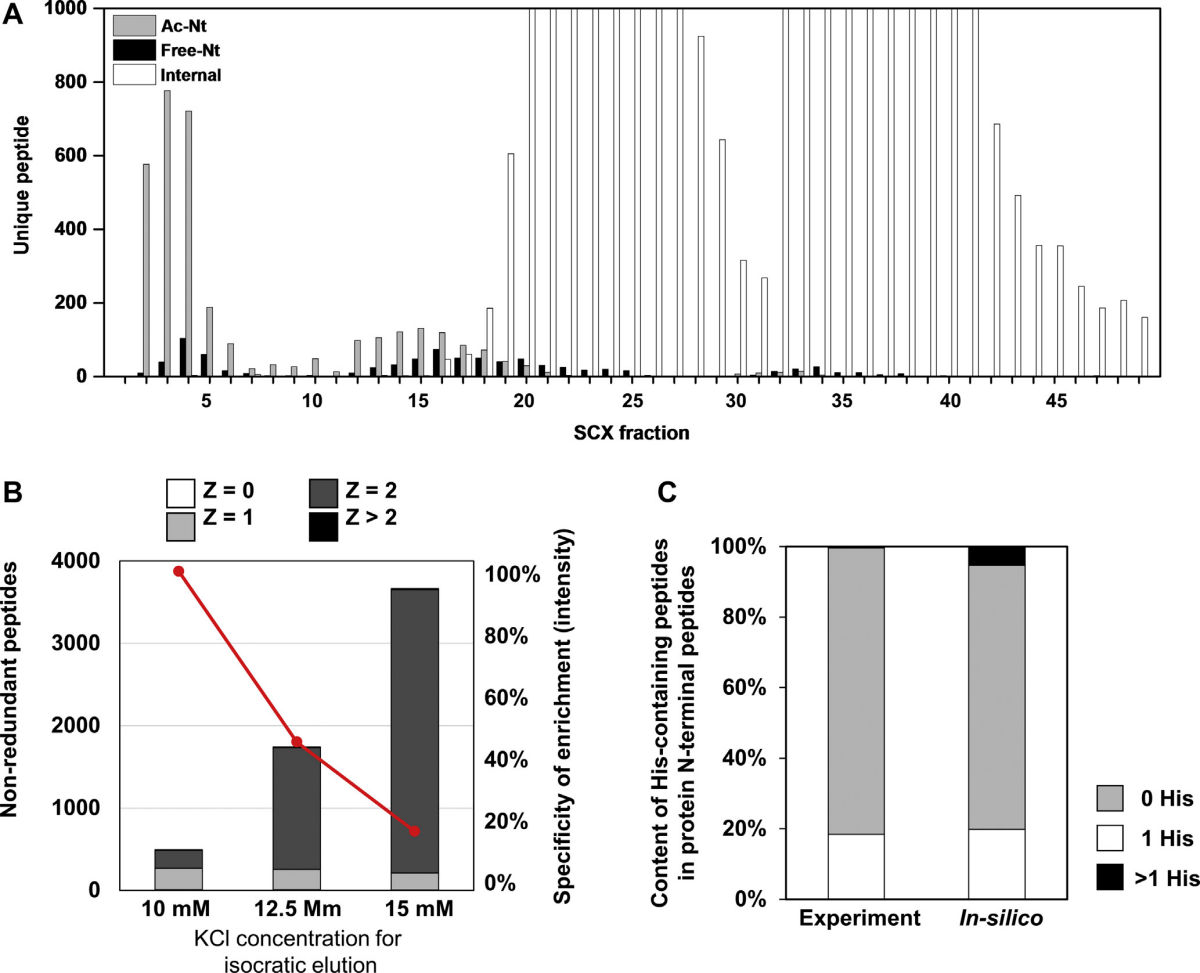
**SCX elution profiles of TrypN-digested peptides**. Z is the charge number at acidic pH, which is based on the number of basic residues per peptide, such as unmodified N terminus, Lys, Arg and His. *A*, SCX HPLC fractionation of different types of peptides in TrypN-digested HEK293T cell lysates using KCl salt gradient elution. In fractions 2 to 6, protein N-terminal peptides with Z = 0 and 1 were observed, such as acetylated protein N-terminal peptides with or without one basic amino acid and unmodified protein N-terminal peptides without basic amino acid. *B*, SCX HPLC separation of TrypN-digested *E. coli* peptides under isocratic conditions with different KCl concentrations. The specificity in enriching protein N-terminal peptides and the numbers of peptides with different Z values are shown. *C*, the content of His-containing peptides in protein N-terminal peptides. The experimental results were those obtained with 10 mM KCl isocratic elution, and the *in-silico* results were calculated from the *E. coli* K-12 MG1665 protein sequence database. Details of *in-silico* digestion are described in the supplemental methods. SCX, strong cation exchange.

The HPLC system used in this study was equipped with a nonporous hydrophilic SCX column having a separation efficiency equivalent to that of a typical reversed-phase column (the peak width at half height was 12.4 ± 4.2 s, and the peak capacity was 122; see supplemental Fig S2). As already shown in Figure 1*A*, this SCX HPLC system was able to separate TrypN-digested peptides with +1 and +2 charges from each other. Comprehensive SCX fractionation of TrypN-digested peptides derived from HEK293T cells was performed with a KCl salt gradient elution at pH 2.6, and the peptide identification for each fraction was performed by nanoLC/MS/MS (supplemental Table S2). As shown in Figure 2*A*, nearly all of the protein N-terminal–derived peptides were clearly separated from the internal peptides, regardless of whether their N termini were acetylated or unmodified. The fractions from 2 to 11 min contained mainly 0 and +1 peptides, including 2207 acetylated protein N-terminal peptides, 345 His-containing acetylated N-terminal peptides, and 262 unmodified N-terminal peptides. The 12- to 18-min fractions contained +2 peptides, *i.e*., unmodified protein N-terminal peptides containing one His, Lys, or Arg and acetylated protein N-terminal peptides containing two basic amino acids. The next fractions from 19 to 30 min also contained +2 peptides, but most of them were internal peptides based on the orientation effect, *i.e.,* retention was stronger because of the high density of positive charge at the N terminus of the peptides (Fig. 1*B*). Thus, the protein N-terminal peptides can be easily isolated. Peptides with a charge greater than 2+ were sequentially eluted in the fractions after 31 min. These included protein N-terminal peptides containing missed cleavage sites, but their number was small because of the high efficiency of TrypN digestion by the PTS method. Up to 90% of nonredundant protein N-terminal peptides could be recovered in fractions up to 18 min by this approach (supplemental Fig. S3), demonstrating that the combination of TrypN digestion with SCX HPLC enables simple and rapid protein N-terminal peptide enrichment. In addition, unlike trypsin, which is unable to cleave Lys-Pro and Arg-Pro bonds, TrypN can cleave Pro-Lys and Pro-Arg bonds, generating protein N-terminal peptides with Pro at the C termini and thus improving the coverage in N terminomics.

Because the charge number of the protein N-terminal peptides at acidic pH is generally smaller than that of internal peptides, we examined whether the identification efficiency of protein N-terminal peptides is affected by the low positive charge number. Because peptide identification is influenced by several steps, including ionization, ion transmission from MS1 to MS2, and fragmentation, four parameters such as the UV absorbance at 214 nm in SCX, the total ion currents in MS and MS/MS scans, and the Mascot peptide score distribution were measured for SCX 1- to 18-min fraction (protein N-terminal peptides were enriched) and 19- to 50-min fraction (internal peptides were enriched), respectively (supplemental Table S3). Considering that the UV absorbance ratio of the 1- to 18-min fraction to the 19- to 50-min fraction was smaller than the ratio of the average total ion current per MS scan, the ionization efficiency of protein N-terminal peptides was better than that of the internal peptides because of the lower sample complexity of the 1- to 18-min fraction. For ion transmission efficiency from MS1 to MS2, we did not see any difference between protein N-terminal peptides and internal peptides. As for fragmentation, the profiles of charge number distribution at acidic pH were significantly different between the protein N-terminal and internal peptides, but the obtained profiles of the score distribution were almost identical. This could be because of the similar distribution profiles of the charge states of the precursor ions. These results indicate that there is no clear disadvantage of using TrypN for the identification of the protein N-terminal peptides.

### Optimization of SCX Separation Using TrypN-Digested *Escherichia coli* Peptides

To optimize the elution conditions for isolation of protein N-terminal peptides, we employed *E. coli* TrypN-digested peptides. Because bacterial proteins have less N-terminal modification than mammalian proteins, the bacterial sample was considered preferable to optimize the conditions for separating the protein N-terminal peptides with a +2 charge (peptides with an unmodified N terminus and one His residue) from the internal peptides (supplemental Fig. S1). Three SCX buffers with different KCl concentrations were used for isocratic elution for 30 min, and the enrichment efficiencies for protein N-terminal peptides were compared (supplemental Table S4). An enrichment specificity of more than 97% was obtained with 10 mM KCl (Table 1). When buffers with higher KCl concentrations were used, more internal peptides were identified (Fig. 2*B*). In the case of 10 mM KCI buffer, we identified 53 His-containing protein N-terminal peptides out of 270 nonredundant protein N-terminal peptides without missed cleavage from 20 μg of *E. coli* lysate (19.6%, Fig. 2*C*). Among *in silico* TrypN-digested peptides from the *E. coli* proteome, 20% of the protein N-terminal peptides contain one His, suggesting that our enrichment conditions have no bias in identifying His-containing protein N-terminal peptides. In other words, this SCX chromatography was able to isolate the protein N-terminal peptides from TrypN-digested *E. coli* peptides even in the most difficult cases, in which the unmodified protein N-terminal peptides contain an additional basic amino acid such as His, Lys, or Arg near the N terminus (supplemental Fig. S4). Although this SCX separation can be explained by the charge/orientation model, it is the first report to apply the retention model to N-unmodified protein N-terminal peptides.

**Table 1:** Enrichment of *E. coli* protein N-terminal peptides by SCX HPLC with isocratic elution at three different KCl concentrations

Salt concentration	10 mM	12.5 mM	15 mM
Unique peptides	432	1669	3416
Unmodified protein N-terminal peptides	326	444	387
Acetylated protein N-terminal peptides	31	25	22
Protein N-terminal peptides (%) based on peptide counts	82.6	28.1	12
Protein N-terminal peptides (%) based on peak area	98.2	49.2	18.5

The enrichment specificity of protein N-terminal peptides is obtained by calculating the number and LC/MS peak area of protein N-terminal peptides among all identified peptides.

SCX, strong cation exchange.

### HEK293T Protein N-Terminal Peptide Enrichment by TrypN-SCX Approach

**Fig. 3:**
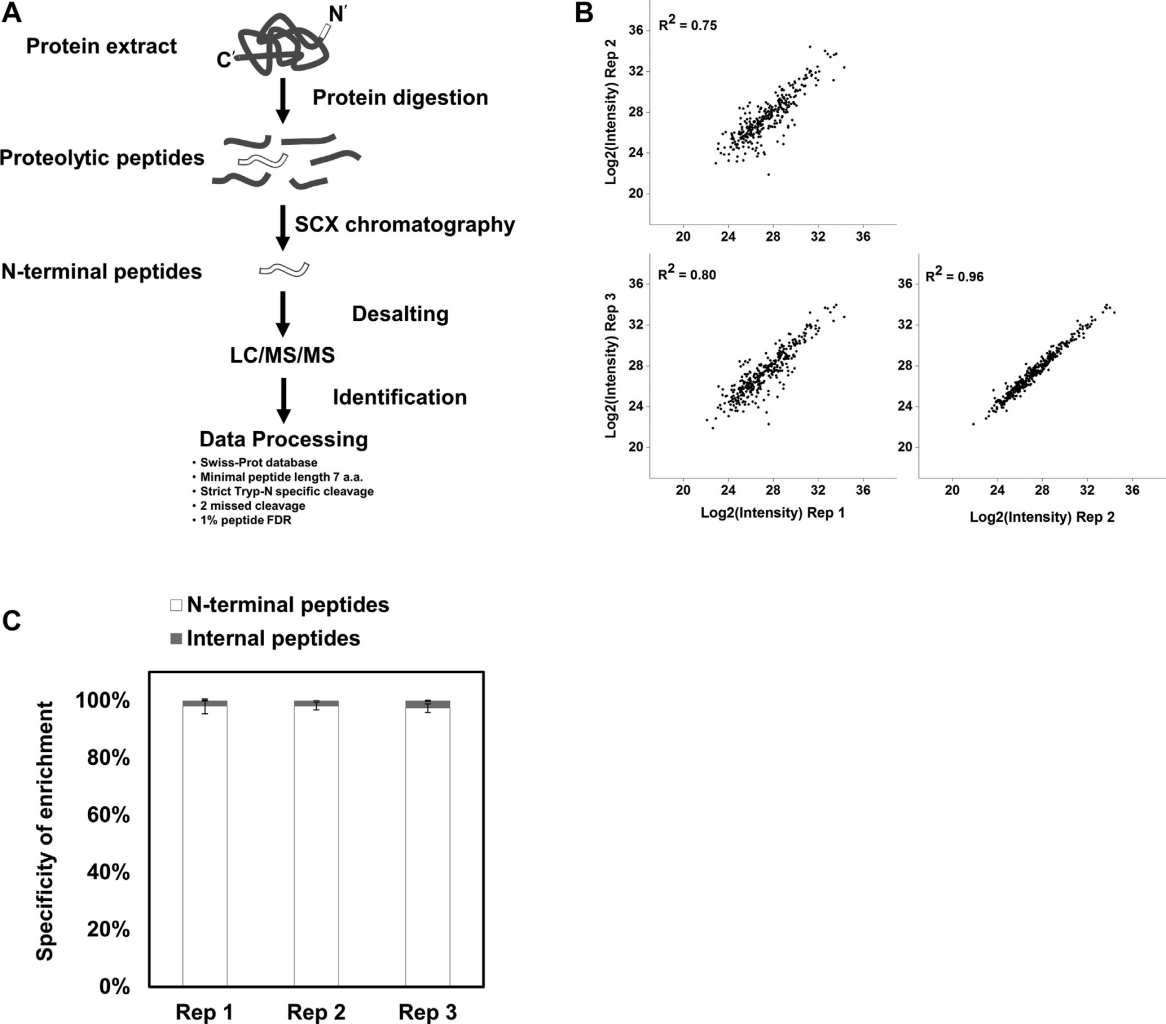
**N-Terminal proteomics using SCX chromatography.***A*, workflow of TrypN/SCX-based N-terminal proteomics. For details, see Experimental Procedures. Three technical replicates were conducted on Day 1 (Rep 1) and Day 2 (Rep 2 and Rep 3) to evaluate interday and intraday reproducibility. *B*, reproducibility in quantifying peptide peak areas between three technical replicates. Rep 1-Rep 2 and Rep1-Rep 3 correlation represent interday reproducibility, whereas Rep 2-Rep 3 correlation shows intraday reproducibility. *C*, enrichment specificity for protein N-terminal peptides in three technical replicates. SCX, strong cation exchange.

The N-terminal peptides of His-containing proteins could be successfully separated from the internal peptides of human and bacterial samples by SCX HPLC under optimized elution conditions. To validate the applicability of this method to large-scale N-terminal proteomics, we performed triplicate analyses using HEK293T cells, which have been widely used in N-terminal proteomics (17, 18). Triplicate SCX HPLC fractionations using 10 mM KCl isocratic elution were done for TrypN-digested HEK293T peptides (80 μg each time), and we subjected one-fourth of the isolated peptides to nanoLC/MS/MS in triplicate (nine runs in total). Default parameters, such as the Swiss-Prot human protein sequence database, specific TrypN cleavage, and minimum peptide length of seven amino acids, were applied for peptide identification by database search (Fig. 3*A*). The results are shown in Figure 3*B*, Table 2, and supplemental Table S5. High correlations of peak areas of identified peptides were observed for intraday and interday preparation samples (R^2^ = 0.96 and R^2^ = 0.75, 0.80, respectively). On average, we identified 1550 unique acetylated and 200 unmodified protein N-terminal peptides from 20 μg of TrypN-digested HEK293T peptides in a single LC/MS/MS analysis. Contamination by internal peptides amounted to only 3% and 9% in peptide peak area and peptide number, respectively (Fig. 3*C*, Table 2). Protein N-terminal peptides with missed cleavage were also enriched in the same elution, and 850 (∼50%) miscleaved unique N-terminal peptides were identified on average, improving the coverage of the N terminome. We identified 1640 acetylated, 106 partially acetylated, and 167 unmodified nonredundant protein N termini. Note that 1600 additional neo-N-terminal peptides were identified when semispecific cleavage at the N terminus was allowed in the data processing, although our purpose in this study was not to find novel proteoforms but to establish a novel approach for N terminomics. Furthermore, to compare our results with two published N-terminome datasets for HEK293T human cells (17, 18), we reanalyzed those datasets under the same conditions without the use of their original customized database or nonspecific cleavage. In terms of the contents of acetylated and unmodified protein N-terminal peptides, all three datasets provided identical results, whereas the content of internal peptides as well as the number of unique peptides varied depending on the approach and the sample amount (supplemental Fig. S5).

**Table 2:** Identification of protein N-terminal peptides from TrypN-digested HEK293T cells

Peptides and protein groups	Replicate 1	Replicate 2	Replicate 3	Total
Unmodified protein N-terminal peptides	199 (±3)	187 (±5)	197 (±3)	352
Acetylated protein N-terminal peptides	1854 (±13)	1301 (±18)	1509 (±15)	2666
Internal peptides	160 (±8)	147 (±3)	232 (±6)	433
N-term ratio (%, peptide counts)	92.8 (±0.3)	91.0 (±0.2)	88.0 (±0.3)	
N-term ratio (%, peptide area)	97.4 (±0.3)	98.0 (±0.5)	97.4 (±0.5)	
Unmodified protein groups	115 (±3)	100 (±4)	116 (±3)	167
Partially acetylated protein groups	36 (±2)	60 (±3)	50 (±2)	106
Acetylated protein groups	1223 (±7)	1000 (±9)	1187 (±16)	1640

Samples were prepared in triplicate (Replicates 1–3) and nanoLC/MS/MS of each sample was conducted in triplicate. Each number in the table is the average of triplicate measurements, and the total number is calculated after merging all results (n = 9) and removing redundancy. The enrichment specificity of protein N-terminal peptides is obtained by calculating the number and peak area of protein N-terminal peptides among all identified peptides.

In conclusion, we have succeeded in developing a new N-terminomics method that does not require chemical reactions. This simple and rapid approach is suitable for high-throughput screening with minimal sample amounts. Our TrypN-SCX N terminomics can enrich protein N-terminal peptides without bias, including peptides containing basic amino acids, with or without N-terminal modifications. We believe our TrypN-SCX approach has great potential for expanding N terminomics. Potential developments include deeper profiling with additional fractionation, the use of customized databases containing predicted protein N termini, the replacement of HPLC with StageTips for SCX separation, and quantification by isotopic labeling.

## Data availability

All LC/MS/MS data that support the findings of this study have been deposited with the ProteomeXchange Consortium via the jPOST partner repository with the dataset identifier (JPST000422/PXD010551) (37).

## Conflict of interest

Authors declare no competing interests.
