# Supplementary material for: Isolation of Acetylated and Unmodified Protein N-Terminal Peptides by Strong Cation Exchange Chromatographic Separation of TrypN-Digested Peptides
Source: Mol Cell Proteomics. 2020 Nov 24;20:100003. doi: 10.1074/mcp.TIR120.002148 (PMC7857546; doi:10.1074/mcp.TIR120.002148)
Supplement: Supplementary file 2 — Supplemental Data [file mmc2.pdf]

SUPPLEMENTAL DATA

Isolation of acetylated and unmodified protein N-terminal peptides by strong cation exchange chromatographic separation of TrypN-digested peptides

Chih-Hsiang Chang[1], Hsin-Yi Chang[1], Juri Rappsilber[2, 3] and Yasushi Ishihama[1]*

*Corresponding author: Yasushi Ishihama, E-mail: yishiham@pharm.kyoto-u.ac.jp

Contents:

1. **Supplemental Method:** Preparation of *in silico* digested *E. coli Protein* N-terminal peptide list.
2. **Supplemental Figures:**
   - Supplemental Figure S1. Charge/orientation-based SCX retention model for TrypN-digested peptides.
   - Supplemental Figure S2. Typical chromatogram of high resolution SCX chromatography employed in this study.
   - Supplemental Figure S3. Accumulation of non-redundant protein groups based on protein N-terminal peptides during SCX elution.
   - Supplemental Figure S4. Dependence of the localization of basic amino acids on isolating protein N-terminal peptides by SCX with 10 mM KCl isocratic elution.
   - Supplemental Figure S5. Comparison of the enrichment efficiency for protein N-terminal peptides from HEK293T cell lysate between published datasets and this study.
3. **Supplemental Tables:**
   - Supplemental Table S1. Identification list of TrypN- and trypsin-mixed HEK293T peptides used in Figure 1A.
   - Supplemental Table S2. Identification list of TrypN-digested HEK293T peptides used in Figure 2A.
   - Supplemental Table S3. Evaluation of the identification efficiency of TrypN-digested HEK293T peptides used in Figure 2A.
   - Supplemental Table S4. Identification list of TrypN-digested *E. coli* peptides used in Table 1, Figure 2B and 2C.
   - Supplemental Table S5. Identification list of TrypN-digested HEK293T peptides used in Table 2 and Figure 3.

Supplemental Method:

Preparation of *in-silico* digested *E. coli Protein* N-terminal peptide list

The list of protein N-terminal peptides obtained by *in silico* TrypN-digestion of *E. coli* proteome was prepared using *E. coli* K-12 MG1665 protein sequence database (4,316 sequences) (1). First, a strict TrypN-specific cleavage at protein N-terminus was performed and missed cleavage was prohibited. Second, peptides shorter than 7 amino acids in length and the redundant sequences were removed. A total of 1,494 protein N-termini were obtained.

Supplemental Figures:

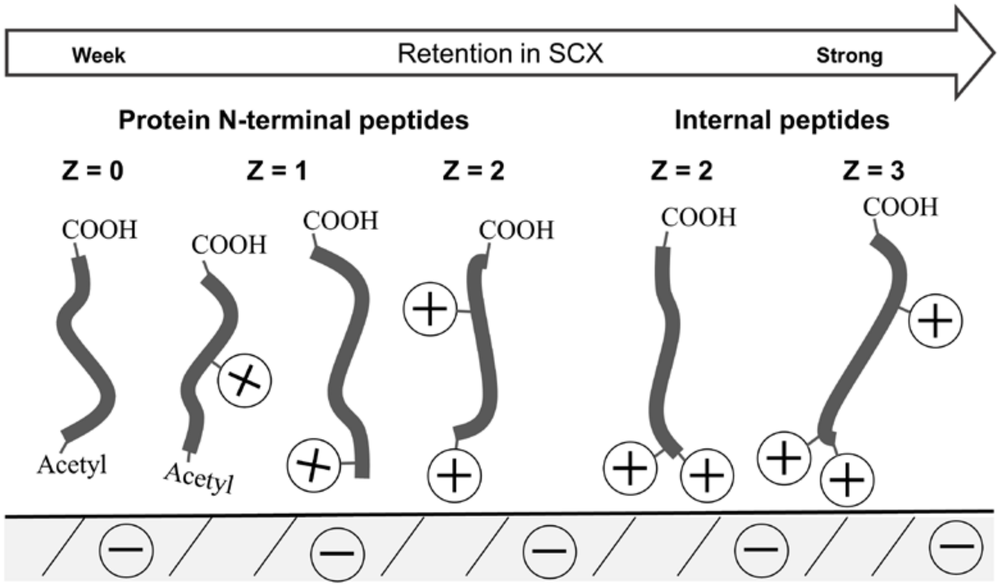

Supplemental Figure S1 Charge/orientation-based SCX retention model for TrypN-digested peptides. Six types of TrypN-digested peptides are shown. Z is the charge number at acidic pH, which is based on the number of basic residues per peptide, such as unmodified N-terminus, Lys, Arg and His.

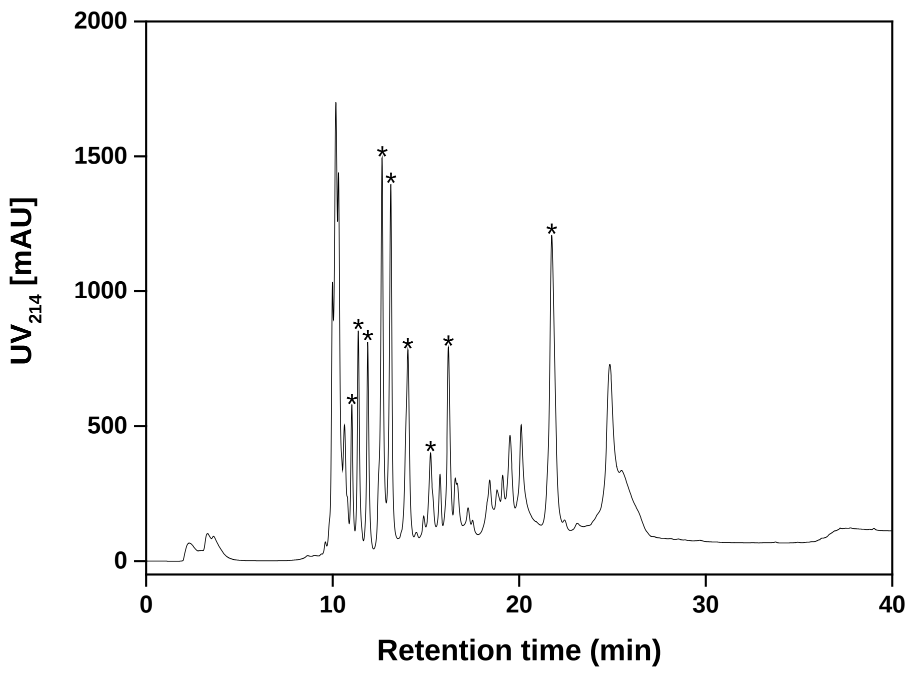

Supplemental Figure S2 Typical chromatogram of high resolution SCX chromatography employed in this study. Tryptic HSA peptides (500 µg) were analyzed by using the Shimadzu Prominence HPLC system with Agilent BioIEX SCX column (250 mm × 4.6 mm inner diameter, 5 µm, non-porous beads made of poly(styrene-divinylbenzene) modified with sulfonate groups). A mixture of 7 mM sodium phosphate (pH 2.6) and ACN (7:3) was used for SCX buffer A, and 1 M NaCl was added to buffer A for SCX buffer B. The two step linear gradient was performed as follows: 0% B for 5 min, 0-40% in 30 min, 40-99% in 5 min and 100% B for 10 min. Peak capacity was calculated using the following equation: $P_C = 1 + t_g/W_{0.5}$. ($W_{0.5}$ = 12.4±4.2 sec, P_C = 122.) Peaks with asterisk were selected for measuring the half peak width ($W_{0.5}$) to calculate peak capacity.

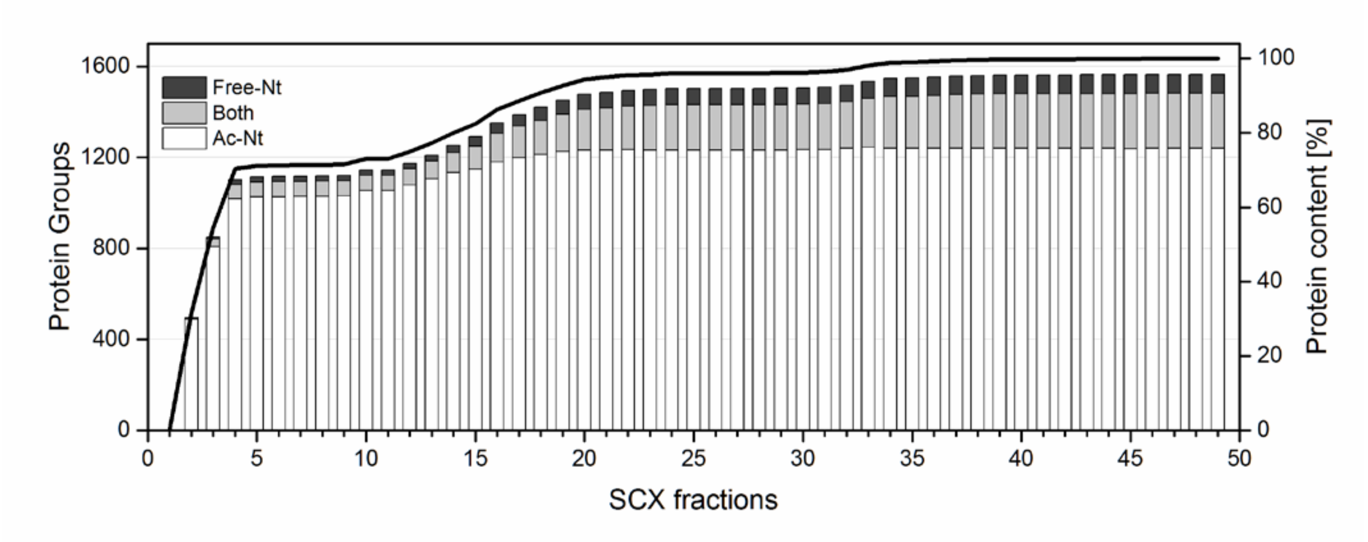

Supplemental Figure S3 Accumulation of non-redundant protein groups based on protein N-terminal peptides during SCX elution. Sample: TrypN-digested HEK293T cell lysate. Free-Nt (black), both (gray) and Ac-Nt (white) squares in the bar graph represent unmodified, partially acetylated and acetylated protein N-termini, respectively. The curve represents the accumulated content of protein groups.

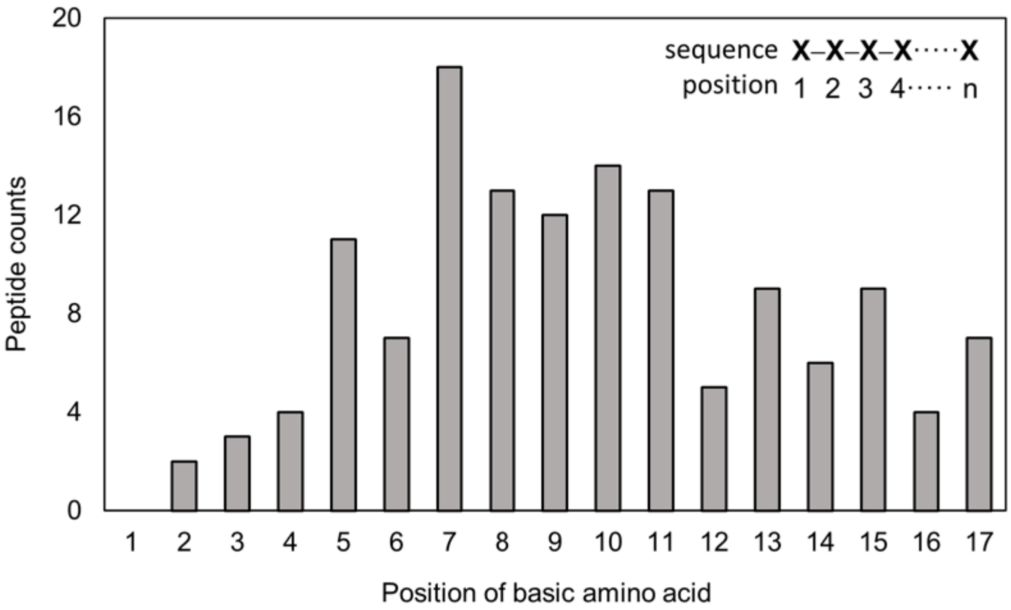

Supplemental Figure S4 Dependence of the localization of basic amino acids (K, R, or H) on isolating protein N-terminal peptides by SCX with 10 mM KCl isocratic elution. Unmodified protein N-terminal peptides with +2 charge (137 peptides) isolated from TrypN-digested *E coli.* cell lysate were plotted.

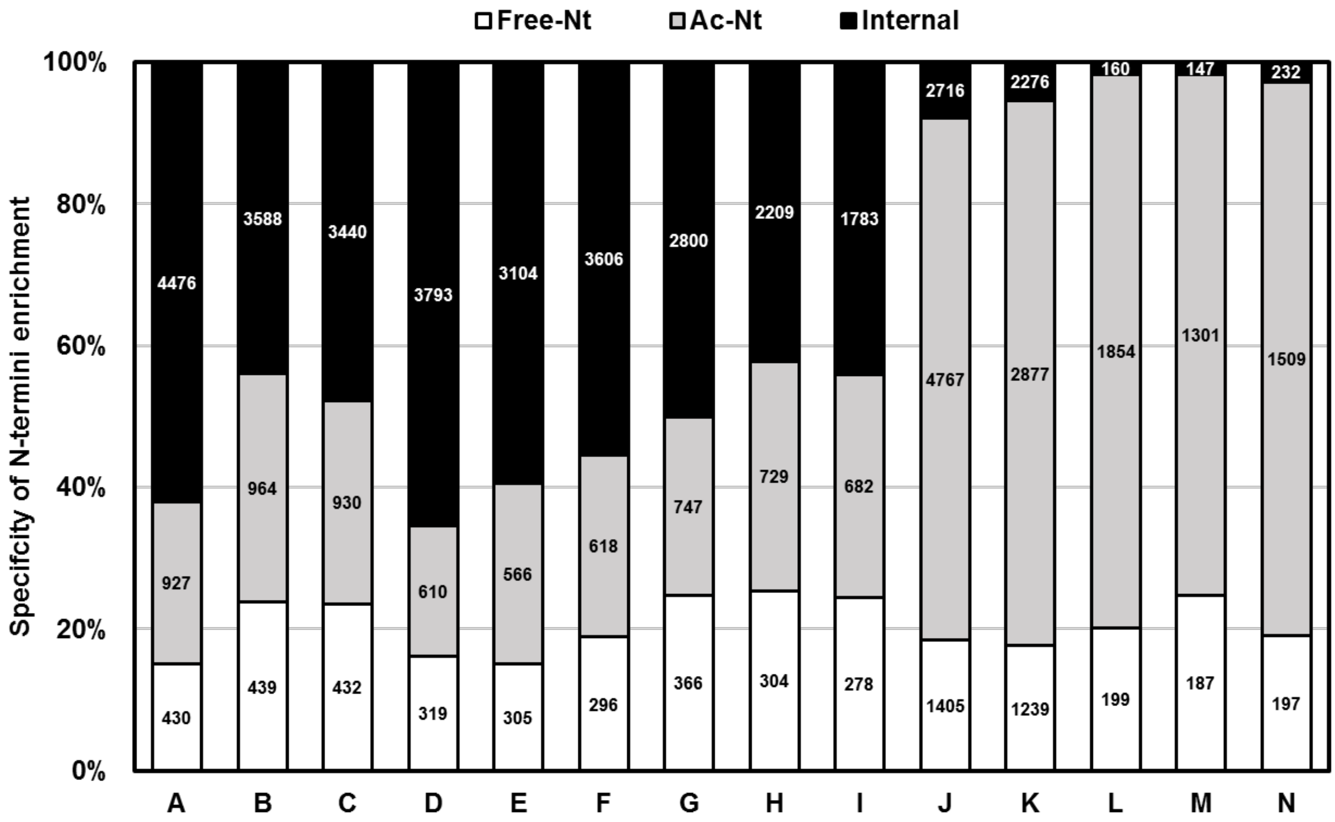

Supplemental Figure S5 Comparison of the enrichment efficiency for protein N-terminal peptides from HEK293T cell lysate between published datasets and this study. The published raw files were download from PRIDE, reanalysis by Mascot 2.6.1 with Swiss-Prot database (version 2017_04, 20,199), and peptides were considered identified if the false discovery rate is set to be less than 1% at peptide level. Specificity was calculated by the peptides area, and the numbers in the bar chart show the number of non-redundant peptides. Entry A-I depleted internal peptides by NHS-beads (2). Entry J and K depleted internal peptides by HPG-ALD (3). Entry L, M and N are the triplicate results in this article. Ac-Nt, Free-Nt and Internal represent acetylated protein N-terminal peptides, unmodified protein N-terminal peptides and internal peptides, respectively.

Supplemental Table S3. Evaluation of the identification efficiency of TrypN-digested HEK293T peptides used in Figure 2A.

	Total peak area in SCX (UV 214 nm)	Average total ion current per MS1 scan	Average total ion current per MS2 scan	Mascot peptide score (average ± SD)	Precursor ion charge state (1+/2+/3+/>3+)	Charge number at acidic pH (0/+1/+2/+3/>+3)
Protein N-terminal peptides (fr1-18)	5.8E+04	1.9E+08	3.0E+06	51 ± 26	1/86/11/2	21/41/37/1/0
Internal peptides (fr19-50)	3.5E+05	9.4E+08	1.5E+07	52 ± 24	0/62/33/4	0/0/52/28/20
Protein N-term/Internal ratio	0.17	0.20	0.20	0.98	-	-

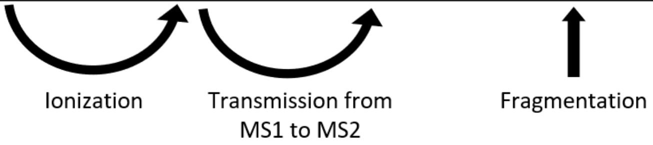

References

1. Riley, M., Abe, T., Arnaud, M. B., Berlyn, M. K. B., Blattner, F. R., Chaudhuri, R. R., Glasner, J. D., Horiuchi, T., Keseler, I. M., Kosuge, T., Mori, H., Perna, N. T., Plunkett, G., Rudd, K. E., Serres, M. H., Thomas, G. H., Thomson, N. R., Wishart, D., and Wanner, B. L. (2006) Escherichia coli K-12: a cooperatively developed annotation snapshot - 2005. *Nucleic Acids Res.* **34**, 1-9

2. Yeom, J., Ju, S., Choi, Y., Paek, E., and Lee, C. (2017) Comprehensive analysis of human protein N-termini enables assessment of various protein forms. *Sci. Rep.* **7**, 6599

3. Na, C. H., Barbhuiya, M. A., Kim, M. S., Verbruggen, S., Eacker, S. M., Pletnikova, O., Troncoso, J. C., Halushka, M. K., Menschaert, G., Overall, C. M., and Pandey, A. (2018) Discovery of noncanonical translation initiation sites through mass spectrometric analysis of protein N termini. *Genome Res.* **28**, 25-36
